# Trained lay health workers reduce common mental disorder symptoms of adults with suicidal ideation in Zimbabwe: a cohort study

**DOI:** 10.1186/s12889-018-5117-2

**Published:** 2018-02-08

**Authors:** Epiphany Munetsi, Victoria Simms, Lloyd Dzapasi, Georgina Chapoterera, Nyaradzo Goba, Tichaona Gumunyu, Helen A. Weiss, Ruth Verhey, Melanie Abas, Ricardo Araya, Dixon Chibanda

**Affiliations:** 1 Zimbabwe AIDS Prevention Project, 92 Prince Edward Road Milton Park, Harare, Zimbabwe; 2 MRC Tropical Epidemiology Group, London School of Hygiene and Tropical Medicine, Keppel Street, London, WC1E 7HT UK; 3 King’s College London, Institute of Psychiatry, Psychology and Neuroscience, De Crespigny Park, London, SE5 8AF UK

**Keywords:** Suicidal ideation, Lay health workers, Common mental disorders

## Abstract

**Background:**

Suicidal ideation may lead to deliberate self-harm which increases the risk of death by suicide. Globally, the main cause of deliberate self-harm is depression. The aim of this study was to explore prevalence of, and risk factors for, suicidal ideation among men and women with common mental disorder (CMD) symptoms attending public clinics in Zimbabwe, and to determine whether problem solving therapy delivered by lay health workers can reduce common mental disorder symptoms among people with suicidal ideation, using secondary analysis of a randomised controlled trial.

**Methods:**

At trial enrolment, the Shona Symptom Questionnaire (SSQ) was used to screen for CMD symptoms. In the intervention arm, participants received six problem-solving therapy sessions conducted by trained and supervised lay health workers, while those in the control arm received enhanced usual care. We used multivariate logistic regression to identify risk factors for suicidal ideation at enrolment, and cluster-level logistic regression to compare SSQ scores at endline (6 months follow-up) between trial arms, stratified by suicidal ideation at enrolment.

**Results:**

There were 573 participants who screened positive for CMD symptoms and 75 (13.1%) reported suicidal ideation at baseline. At baseline, after adjusting for confounders, suicidal ideation was independently associated with being aged over 24, lack of household income (household income yes/no; adjusted odds ratio 0.52 (95% CI 0.29, 0.95); *p* = 0.03) and with having recently skipped a meal due to lack of food (adjusted odds ratio 3.06 (95% CI 1.81, 5.18); *p* < 0.001). Participants who reported suicidal ideation at enrolment experienced similar benefit to CMD symptoms from the Friendship Bench intervention (adjusted mean difference − 5.38, 95% CI −7.85, − 2.90; *p* < 0.001) compared to those who had common mental disorder symptoms but no suicidal ideation (adjusted mean difference − 4.86, 95% CI −5.68, − 4.04; *p* < 0.001).

**Conclusions:**

Problem-solving therapy delivered by trained and supervised lay health workers reduced common mental disorder symptoms among participants with suicidal thoughts who attended primary care facilities in Zimbabwe.

**Trial registration:**

pactr.org ldentifier: PACTR201410000876178

## Background

Suicide is a severe public health problem [1], which is common in both males and females, especially males [2]. However nine independent epidemiological surveys, a cohort study in Vietnam and a national survey in the UK found that suicidal thoughts were more common in women and 16 to 24 year olds [3–5]. A wide range of mental disorders increase the risk of suicidal ideation [6], also known as suicidal thoughts. Globally, depression contributes significantly to suicidal ideation [7], which increases the risk of death by suicide [8]. The estimated number of suicidal deaths related to mental disorders has increased from 138,000 in 1990 to 232,000 in 2010 [9].

Suicide data from the southern African region are limited as data on deliberate self-harm are often not recorded [10]. In a cross-sectional survey of 842 women attending 6-week postpartum clinics in Harare, Zimbabwe, 21.6% reported suicidal ideation postpartum [11]. Being unmarried, widowed, divorced or separated increased the risk of suicide [4, 12]. A cohort study in New Zealand discovered that being unemployed was associated with a higher risk of suicidality [13].

HIV status can contribute to risk of suicidal behaviour, as shown by a systematic review [14]. People living with HIV have a higher prevalence of suicidal ideation than those who are HIV negative [15], however suicidal thoughts have decreased considerably as a result of the introduction of HAART [16]. Both of these studies were conducted in high-income settings (the USA and Switzerland).

Mental, neurological and substance use disorders contribute significantly to the global burden of disease [17]. In low and middle income countries many people suffer from these conditions [18] but over 75% do not get treatment due to lack of economic resources and mental health services [19] and professionals [20]. Treatment coverage ranges from 10 to 90% in these settings [21].

The patient-to-psychiatrist ratio on average in low income countries is 1:1.7 million [22]. To address this treatment gap, interventions have been developed that tackle common mental disorders (CMDs) through task sharing to lay health workers (LHWs) [23]. Task sharing is the delegation of responsibilities to lower level cadres who are supported and supervised by more senior professionals [24].

A lay health worker may be any health worker offering services related to health care delivery who has no formal professional training but has received some basic training [25]. LHWs are trained to provide selected health services allowing more highly trained workers to handle more complex tasks for which specialist training is required [26]. Additionally, LHWs have in-depth knowledge of a community and culture, which may make them better equipped to handle certain health challenges [27].

Interest in LHW programmes has increased [25], as they can assist in HIV service delivery [28]. Evidence suggests that non specialist workers are capable of providing counselling and case management at community level [29]. A systematic review from a number of health care facilities show that positive health results for patients with HIV can be attained by task sharing that involves LHWs [30].

Mental health care can be delivered effectively through the use of trained and supervised LHWs [31, 32]. A Cochrane review found that the use of LHWs to deliver mental health interventions may result in positive treatment outcomes for patients with common mental disorders (CMDs) [33]. A study in India concluded that trained LHWs within a collaborative care model can reduce prevalence of CMDs among those attending public primary care facilities [34].

In a recently-completed cluster-randomised controlled trial, a LHW intervention at primary care level in Zimbabwe, the Friendship Bench LHW programme, was effective in reducing CMD symptoms, disability, and improving quality of life [35, 36]. The prevalence of suicidal ideation after 6 months was 2.3% in the intervention group versus 12.3% in the control group, from a baseline of 11–13% [36]. While the Friendship Bench was effective in reducing suicidal ideation, it is not known whether participants with suicidal ideation (which may be more difficult for LHWs to manage) benefitted as much from the intervention as other participants.

The objectives of this study were (1) to explore prevalence of, and risk factors, for suicidal ideation among men and women with CMD symptoms attending public clinics in Zimbabwe at enrolment to the Friendship Bench trial, and (2) to determine whether problem-solving therapy by LHWs can reduce symptoms of common mental disorders among people with suicidal ideation.

## Methods

### Study design

We previously conducted a cluster-randomized controlled trial of the Friendship Bench LHW counselling intervention. The trial took place in 24 Harare city health primary care clinics, 12 randomly allocated to the intervention and 12 control clinics delivering enhanced standard care. Primary care clinics in Harare offer family health services, opportunistic infection treatment, and treatment of other physical non-complicated medical cases.

Participants in the intervention arm received up to six sessions of one-to-one counselling from a LHW and linkages to an optional peer support group with an income generation component. The intervention has previously been described in detail [36]. The counselling involved problem-solving therapy, teaching participants a structured approach to identifying problems and workable solutions. The six sessions were usually completed within 3 weeks after enrolment. Participants at the enhanced standard care clinics received the usual care plus support and information on common mental disorders, including assessment for antidepressant medication or referral to a psychiatric facility as needed.

### Study sample

For 2 weeks per clinic, people attending the clinic for any reason were randomly selected based on their number in the queue. Those who were aged 18 years and above, residing within the clinic’s geographic area, in possession of a valid national identification card for age verification and willing to give consent were screened using the Shona Symptom Questionnaire (SSQ14). The SSQ measures symptoms of common mental disorders, and was developed in Zimbabwe and revalidated for the study population, with 84% sensitivity and 73% specificity against a diagnosis of depression and/or anxiety [37]. Participants who scored above 9/14 on the SSQ14 and answered yes to a question about suicide thoughts in the past week which was one of the 14 items were designated suicidal and also had CMD symptoms [36].

Patients were excluded from the study if they were considered too ill; pregnant and in their third trimester or 3 months post-partum; did not have a traceable address or a working phone; did not understand English or Shona; had suicidal intent; were in psychiatric care; presented with psychosis, intoxication or dementia; or were not willing to have home visits by study staff members. Individuals with suicidal ideation but not subsequently assessed as having suicidal intent were eligible to participate.

The SSQ score and other outcomes for each participant were collected at baseline and at 6 months after the day of recruitment.

### Data analysis

Data were exported to Stata 14.1 for analysis. The association between suicidality at baseline and other baseline factors was estimated using logistic regression, adjusting for clinic as a random effect. All variables associated with the outcome at a significance level of *p* < 0.2 were carried forward into multivariate analysis, plus age and gender as a priori independent variables. Variables were assessed for collinearity by comparison with the univariate results and removed as necessary. Mean and 95% confidence intervals of SSQ score were calculated at baseline and follow-up by baseline suicidality and arm, adjusting for clustering. Analysis of the effect of the intervention was based on cluster-level summary measures because of the small number of clinics per arm. The difference in mean SSQ scores between arms at 6 months was estimated using linear regression adjusted for HIV status, sex, baseline score, age and education, as predetermined in the trial analytical plan. In this paper, as an exploratory analysis, we examined the intervention effect stratified by suicidal ideation at baseline.

## Results

A total of 573 participants screened positive for CMD symptoms and took part in the trial, 286 in the intervention arm and 287 in the control arm, the majority of whom (86%) were female (Table 1). The most commonly reported reasons for attending the clinic were bringing a sick relative (40%) or HIV care (20%). Of the men in the sample, 29 (37%) earned a salary, 24 (31%) were self-employed or casually employed, and 25 (32%) were not working.

**Table 1:** Characteristics of participants by suicidal ideation

		No suicidal ideation		Suicidal ideation	
		*N*	%	*N*	%
	Total	498	100.0	75	100.0
Gender	Male	71	91	7	9
	Female	427	86.3	68	13.7
Age	18–24	100	93.5	7	6.5
	25–34	182	83.1	37	16.9
	35–44	132	86.8	20	13.2
	45+	83	88.3	11	11.7
Earning salary	No	272	85.3	47	14.7
	Yes	224	88.9	28	11.1
Marital status	Married/cohabiting	335	86.8	51	13.2
	Divorced/separated/widowed	134	86.5	21	13.5
	Single	28	90.3	3	9.7
Children aged < 16 at home	0	89	85.6	15	14.4
	1	115	86.5	18	13.5
	2	133	86.9	20	13.1
	3	96	87.3	14	12.7
	4+	63	88.7	8	11.3
HIV status	Positive	199	83.6	39	16.4
	Negative	229	88.1	31	11.9
	Declined/missing	68	93.2	5	6.8
Household has an income	Yes	408	88.5	53	11.5
	No	75	78.9	20	21.1
Chronic condition	Yes	122	87.1	18	12.9
	No	374	86.8	57	13.2
Skipped at least one meal in the past month due to lack of food	Yes	182	79.1	48	20.9
	No	314	92.1	27	7.9
Went to sleep hungry in the past week	Yes	120	80.0	30	20.0
	No	376	89.3	45	10.7

Of the 573 participants, 75 (13.1%, 95% CI 10.4–16.1) reported suicidal ideation in the past week at baseline (female = 68; male = 7). At baseline the age range of 25–34 year olds had the highest prevalence of suicidal thoughts (16.9%). Participants who earned a salary were less at risk of suicidal ideation (11.1%) as compared to the ones who did not (14.7%) but the difference was not statistically significant. Of the participants whose household did not have an income 21.1% had suicidal ideation as compared to 11.5% of those who had an income (*p* = 0.01).

Participants who had separated with their spouses through divorce or widowing had almost the same probability of suicidal ideation with 13.5% as the ones who were married or cohabiting (13.2%). Participants living with HIV had slightly higher prevalence of suicidal ideation (16.4%) than the ones who were HIV negative (11.9%). A fifth (20.9%) of participants who had skipped at least one meal in the past month because there was not enough food reported suicidal ideation, compared to7.9% of participants without food insecurity (*p* < 0.01).

In univariate analysis suicidal ideation at baseline was associated with age, HIV status, skipping meals due to lack of food, going to sleep hungry, and lack of household income (Table 2). In a multivariate model, risk factors for suicidal ideation were skipping meals, lack of household income and age. Hunger did not remain in the model due to collinearity with skipping meals.

**Table 2:** Factors associated with suicidal ideation at baseline

		Univariate		Multivariate	
		OR (95% CI)	*p*	OR (95% CI)	*p*
Gender (reference: male)	Female	1.62 (0.71, 3.70)	0.25	1.34 (0.57, 3.15)	0.50
Age (reference: 18–24)	25–34	2.90 (1.25, 6.77)	0.09	3.00 (1.26, 7.16)	0.09
	35–44	2.15 (0.87, 5.31)		2.30 (0.91, 5.82)	
	45+	1.88 (0.69, 5.10)		1.94 (0.69, 5.49)	
Earning salary (reference: no)	Yes	0.73 (0.44, 1.20)	0.21		
Marital status (reference: married)	Divorced/ separated/ widowed	1.02 (0.59, 1.77)	0.95		
	Single	0.70 (0.20, 2.39)	0.57		
Children aged < 16 at home (reference: 0)	1	0.94 (0.44, 1.98)	0.87		
	2	0.90 (0.43, 1.86)	0.77		
	3	0.88 (0.40, 1.94)	0.75		
	4+	0.75 (0.30, 1.89)	0.55		
HIV status (reference: negative)	Positive	1.47 (0.88, 2.47)	0.14		
	Declined/ missing	0.54 (0.20, 1.45)	0.22		
Has any household income (reference: no)	Yes	0.49 (0.27, 0.87)	0.01	0.52 (0.29, 0.95)	0.03
Chronic condition (reference: no)	Yes	0.97 (0.55, 1.72)	0.92		
Skipped at least one meal in the past month due to lack of food (reference: no)	Yes	3.07 (1.85, 5.09)	< 0.001	3.06 (1.81, 5.18)	< 0.001
Went to sleep hungry in the past week (reference: no)	Yes	2.09 (1.25, 3.47)	0.01		

At 6-months follow-up, severity of CMD symptoms were significantly less among participants who received LHW Friendship Bench care than among participants in the control arm. This difference was similar among those who had suicidal ideation at baseline, and those who did not (Table 3). The adjusted mean difference in SSQ-14 score among participants with suicidal ideation was − 5.38 (95% CI −7.85, − 2.90; *p* < 0.001) and among those with common mental disorder symptoms but no suicidal ideation the adjusted mean difference was − 4.86 (95% CI −5.68, − 4.04; *p* < 0.001).

**Table 3:** Effect of the Friendship Bench intervention on CMD symptoms at 6 months, by suicidal ideation at baseline

	Mean SSQ-14 score (95% CI)		Unadjusted mean difference (95% CI), *p*	Adjusted^a^ mean difference (95%CI), *p*
	Intervention arm	Control arm		
Suicidal ideation at baseline	3.28 (1.67, 4.89)	9.82 (8.02, 11.61)	−6.54 (−8.55, −4.53), *p* < 0.001	−5.38 (−7.85, −2.90), *p* < 0.001
No suicidal ideation at baseline	3.74 (3.11, 4.38)	8.76 (8.22, 9.30)	−5.02 (− 5.86, − 4.18), *p* < 0.001	− 4.86 (− 5.68, − 4.04), *p* < 0.001

^a^ Adjusted for age, sex, HIV status, SSQ-14 core at baseline, and education

## Discussion

The aim of the study was to explore the prevalence of suicidal ideation among men and women with CMD symptoms attending public clinics in Harare and to determine whether the Friendship Bench LHW programme can reduce common mental disorder symptoms among people with suicidal ideation. The results show that suicidal ideation is high in this population, especially among women. This outcome supports evidence from the literature that prevalence of suicidal thoughts is highest in females [3]. Participants from households without an income had twice the odds of suicidal ideation compared to those from households with an income. Similarly participants with insufficient food for all meals had almost three times the odds of suicidal ideation. This study confirms existing evidence on the relationship between food insecurity and mental health [38].

The study found that 18–24 year olds had the lowest prevalence of suicidal thoughts, contrary to a study conducted in Vietnam in the general population [5], which found the highest prevalence in people aged 16 to 24. The study did not show a statistically significant association between earning a salary and suicidality. This finding does not align with a cohort study in New Zealand which showed a close relationship between unemployment and suicidality in the general population [13], but in this context it could be because the Friendship Bench participants mainly constituted of women with young children who could probably have been depending financially on their husbands or staying at home to be with their children. Instead, lack of household income rather than lack of personal income was a risk factor for suicidal ideation. In this mainly female population, those at increased risk of suicidal thoughts appear to be women of the age most likely to be caring for young children, whose partners were out of work, and who did not have enough to eat. The unemployment rate in Zimbabwe is extremely high, as shown in this sample where 32% of men were unemployed and 31% had possibly insecure income.

Widowed, divorced or separated individuals are at increased risk of suicidal ideation [15]. This is contrary to this study which found that participants in this category were not at high risk of suicidal thoughts as compared to the married or cohabiting individuals and this could be attributed to financial constraints on families.

## Limitations

The study has several limitations. Men are underrepresented in this study. Only 78 men out of a total of 573 participants took part in this study. The reason why men were few could be that there is always low uptake of health programmes by men and when they report to a health institution they express the need to attend to other commitments. This aligns with a study conducted in Australia which highlighted that young men would prefer to assist themselves than seek professional aid [39]. This could be attributed to the stigma [40] associated with attending a health facility as well as the need to maintain their ego. Reaching men remains a major priority.

## Conclusion

Among clinic attenders in Harare, those aged over 25 with no household income and food insecurity are at increased risk of suicidal ideation. Trained LHWs in primary care clinics can reduce common mental disorder symptoms among people with suicidal ideation in Harare. This finding supports the effectiveness of task shifting from professional health personnel so as to meet individuals at their point of need in primary health care facilities as far as common mental disorders are concerned.

## Abbreviations

CMDs: Common mental disorders
LHWs: Lay health workers
SSQ: Shona Symptom Questionnaire
